# Periodontitis and Cardiovascular Diseases. Consensus Report

**DOI:** 10.5334/gh.400

**Published:** 2020-02-03

**Authors:** M. Sanz, A. Marco del Castillo, S. Jepsen, J. R. Gonzalez-Juanatey, F. D’Aiuto, P. Bouchard, I. Chapple, T. Dietrich, I. Gotsman, F. Graziani, D. Herrera, B. Loos, P. Madianos, J. B. Michel, P. Perel, B. Pieske, L. Shapira, M. Shechter, M. Tonetti, C. Vlachopoulos, G. Wimmer

**Affiliations:** 1 Department of Dental Clinical Specialties and ETEP Research Group, Faculty of Odontology, University Complutense of Madrid, Plaza Ramon y Cajal, Madrid, ES; 2 Cardiology Department, Hospital Universitario Ramon y Cajal, Madrid, ES; 3 Department of Periodontology, Operative and Preventive Dentistry, University of Bonn, Bonn, DE; 4 Cardiology Department, University Hospital, University of Santiago de Compostela, IDIS, CIBERCV, ES; 5 Department of Periodontology, Eastman Dental Institute and Hospital, University College London, London, UK; 6 U.F.R. d’odontologie, Université Paris Diderot, Hôpital Rothschild AP-HP, Paris, FR; 7 School of Dentistry, Institute of Clinical Sciences, College of Medical and Dental Sciences, The University of Birmingham, Birmingham, UK; 8 Heart Institute, Hadassah University Hospital, Jerusalem, IL; 9 Department of Surgical, Medical and Molecular Pathology and Critical Care Medicine, University of Pisa, Pisa, IT; 10 ACTA University, Amsterdam, NL; 11 Department of Periodontology, School of Dentistry, National and Kapodistrian University of Athens, GR; 12 Inserm Unit 1148, laboratory for translational CV science, X. Bichat hospital, Paris, FR; 13 World Heart Federation, Geneva, CH; 14 Centre for Global Chronic Conditions, London School of Hygiene and Tropical Medicine, UK; 15 Charité Universitätsmedizin Berlin, Department of Internal Medicine and Cardiology, Berlin, DE; 16 DZHK (German Center for Cardiovascular Research) Partnersite Berlin, German Heart Institut Berlin, DE; 17 Department of Periodontology, Hebrew University – Hadassah Faculty of Dental Medicine, Jerusalem, IL; 18 Leviev Heart Center, Chaim Sheba Medical Center, tel Hashomer and the Sackler Faculty of Medicine, Tel Aviv University, IL; 19 Department of Periodontology, The University of Hong Kong, Prince Philip Dental Hospital, HK; 20 Department of Cardiology, National and Kapodistrian University of Athens, GR; 21 Department of Prosthetic Dentistry, School of Dental Medicine, Karl-Franzens University Graz, AT

**Keywords:** Periodontitis, periodontal therapy, cardiovascular diseases, chronic inflammation, bacteremia, atherosclerosis, antitrombotic therapy

## Abstract

**Background:**

In Europe cardiovascular disease (CVD) is responsible for 3.9 million deaths (45% of deaths), being ischaemic heart disease, stroke, hypertension (leading to heart failure) the major cause of these CVD related deaths. Periodontitis is also a chronic non-communicable disease (NCD) with a high prevalence, being severe periodontitis, affecting 11.2% of the world’s population, the sixth most common human disease.

**Material and Methods:**

There is now a significant body of evidence to support independent associations between severe periodontitis and several NCDs, in particular CVD. In 2012 a joint workshop was held between the European Federation of Periodontology (EFP) and the American Academy of Periodontology to review the literature relating periodontitis and systemic diseases, including CVD. In the last five years important new scientific information has emerged providing important emerging evidence to support these associations.

**Results and Conclusions:**

The present review reports the proceedings of the workshop jointly organised by the EFP and the World Heart Federation (WHF), which has updated the existing epidemiological evidence for significant associations between periodontitis and CVD, the mechanistic links and the impact of periodontal therapy on cardiovascular and surrogate outcomes. This review has also focused on the potential risk and complications of periodontal therapy in patients on anti thrombotic therapy and has made recommendations for dentists, physicians and for patients visiting both the dental and medical practices.

## 1. Introduction

Non-communicable diseases (NCDs) are rising in prevalence globally in line with an increasingly ageing population, refined diets and sedentary lifestyles. NCDs account for 41 million deaths each year, or 71% of all global deaths [40]. Approximately 80% of people over the age of 65 in the USA are affected by one or more NCDs, and 77% exhibit at least two NCDs, creating a significant burden of disease to individuals and to the healthcare economy [13]. The co-morbid presence of two or more NCDs presents a major challenge to the economy, equating to two thirds of all health costs in the USA [14]; however, less than 1% of health expenditure in the USA is focussed on prevention to improve overall health [129].

The greatest global NCD burden arises due to cardiovascular disease (CVD), which is responsible for 17.9 million deaths (one third of total mortality) and 45% of NCD-induced mortality [104]. In Europe, CVD is responsible for 3.9 million deaths (45% of deaths), and whilst CVD mortality rates are reducing, the absolute numbers have increased in the last 25 years due to an increasingly ageing population [135]. Ischaemic heart disease, stroke, hypertension (leading to heart failure), rheumatic heart disease, cardiomyopathy and atrial fibrillation cause over 95% of CVD-related deaths [103].

In this consensus report, the term CVD is used as a general term for atherosclerotic diseases, principally coronary heart disease, cerebrovascular disease and peripheral vascular disease. A number of chronic infectious-inflammatory-immune diseases are associated with significantly higher risks of adverse cardiovascular events, including rheumatoid arthritis, psoriasis, systemic lupus erythematosus and periodontitis [103], consistent with the thesis that chronic elevations in the systemic inflammatory burden are causally related to CVD development and its sequelae. Whilst there is evidence for over 50 gene polymorphisms playing a role in the modulation of atherogenesis [46], effect sizes are small and the major traditional risk factors for CVD remain lifestyle factors, principally tobacco smoking, dyslipidaemia, hypertension and altered glucose metabolism. The latter correlate strongly with diets high in saturated fats, salt and refined sugars and contribute to obesity and type 2 diabetes mellitus, major attributable risk factors for myocardial infarction [50]. The same risk factors account for over 90% of the stroke burden [87], yet all are modifiable through improved lifestyles, including reducing salt, saturated fat and refined carbohydrate intake, exercising, increasing intake of antioxidant micronutrients and regular moderate alcohol consumption [50].

Periodontitis is also an NCD with a high prevalence of 45–50% overall, with the most severe form affecting 11.2% of the world’s population, making it the sixth most common human disease [52]. The Global Burden of Diseases, Injuries, and Risk Factors Study (2017) of Years Lost to Disability (YLD) reported that from 1990 to 2017 oral diseases (mainly periodontitis and caries) contributed the most YLD in age-standardised prevalence rates from 354 diseases and injuries across 195 countries [39]. There is now a significant body of evidence to support independent associations between severe periodontitis and several NCDs, including diabetes [17], cardiovascular disease [128], chronic obstructive pulmonary disease [64] and chronic kidney disease (CKD) [117]. Indeed, severe periodontitis is independently and significantly associated with all-cause and cardiovascular mortality in several different populations [63,117]. Proposed mechanisms include bacteraemia and the associated systemic inflammatory sequelae, including elevations in C-reactive protein and oxidative stress [113]. In populations with multi-morbidity, for example chronic kidney disease with co-morbid diabetes and periodontitis, periodontitis is associated with significantly reduced survival from all-cause and cardiovascular mortality [117]. It appears, therefore, that periodontitis may be a modifiable non-traditional risk factor for CVD.

In 2012 a joint workshop was held between the European Federation of Periodontology (EFP) and the American Academy of Periodontology to review the literature relating periodontitis and systemic diseases, including CVD. The consensus report was based upon four technical papers that systematically reviewed the evidence for epidemiological associations between periodontitis and incident CVD [28], mechanisms of biological plausibility relating to periodontal bacteria and systemic inflammation [101,113] and periodontal intervention studies [23]. The workshop concluded there was consistent and strong epidemiological evidence that periodontitis imparts increased risk for future atherosclerotic cardiovascular disease. It also concluded the impact of periodontitis on CVD was biologically plausible, via translocated circulating oral microbiota, which may directly or indirectly induce systemic inflammation that impacts upon the development of atherothrombogenesis, and whilst in vitro, pre-clinical and clinical studies supported the interaction and associated biological mechanisms, intervention trials were not sufficiently adequate to draw further conclusions at that time.

The present workshop was jointly organised by the EFP and the World Heart Federation (WHF) to include global experts in both periodontal and cardiovascular disciplines and was held in Madrid on 18 and 19 February 2019. Four technical reviews updating the evidence base from the 2012 workshop were prepared and supplemented by additional studies discussed at the workshop. The reviews focussed on epidemiological associations [45], mechanistic links [114], results from intervention studies [89] and the potential risk and complications of periodontal therapy in patients undertaking antithrombotic (antiplatelet and anticoagulant) therapy.

While this consensus report focuses predominantly on relevant evidence published since the 2012 workshop, there are biological areas that have subsequently come to prominence, and where the underpinning body of evidence was not covered in the 2013 consensus report; hence, certain pre-2012 manuscripts are referenced to ensure the context of these recent studies is clear.

Furthermore, sections 4.3: *What is the effect of statin intake on clinical periodontal outcomes?* and section 5: *Cardiovascular risks and complications of periodontal therapeutic interventions* were not dealt with in the previous workshop; hence, a full appraisal of the scientific evidence was carried out in this consensus meeting.

Finally, following the review of the presented evidence, recommendations for both medical and dental teams, as well as patients and the public, were elaborated.

## 2. Epidemiologic evidence on the association between periodontitis and CVD

### 2.1. Do people with periodontitis have a higher prevalence of subclinical cardiovascular disease?

There is evidence from epidemiological studies that periodontitis patients exhibit significant endothelial dysfunction, measured by flow mediated dilation (FMD), arterial stiffness (e.g., pulse wave velocity – PWV) and a significantly greater thickness of the carotid intima media (cIMT) and elevated arterial calcification scores. There is one imaging study (ATHEROREMO-IVUS study) associating high levels of antibodies against periodontal pathogens and a lower extent of positive atheromatous plaque remodelling [24].

### 2.2. Do people with periodontitis have a higher prevalence of coronary artery disease and risk of myocardial infarction and other coronary events?

There is robust evidence from epidemiological studies for a positive association between periodontitis and coronary heart disease. A systematic review [28], which was updated in preparation for this workshop, identified a total of six case-control and cohort epidemiological studies, published in the last five years, which demonstrated an increased risk of a first coronary event in patients with clinically diagnosed periodontitis or more severe periodontitis compared to patients without periodontitis or less severe periodontitis. Relative risk estimates vary between studies, depending on population characteristics and periodontitis case definitions. There are two cohort studies reporting an association between periodontitis and higher cardiovascular mortality (due to coronary heart disease and cerebrovascular disease).

### 2.3. Do people with periodontitis have a higher prevalence of cerebrovascular disease and risk of stroke?

There is evidence from epidemiologic studies for a positive association between periodontitis and cerebrovascular disease. A systematic review [28], which was updated in preparation for this workshop, identified a total of three case-control and cohort studies, which demonstrate an increased risk of a first cerebrovascular event in patients with clinically diagnosed periodontitis or more severe periodontitis compared to patients without periodontitis or less severe periodontitis. Relative risk estimates vary between studies, depending on population characteristics and periodontitis case definitions. Furthermore, a recent analysis of data from the Atherosclerosis Risk in Communities (ARIC) study demonstrated an association between periodontal profile class and incident ischemic stroke. In this cohort, patients with periodontitis had more than double the risk of cardioembolic and thrombotic stroke compared to periodontally healthy individuals [115]. In addition, as previously documented, there are two cohort studies reporting an association between periodontitis and higher cardiovascular mortality (due to coronary heart disease and cerebrovascular disease) [28].

### 2.4. Do people with periodontitis have a higher prevalence and incidence of Peripheral Artery Disease (PAD)?

There is limited but consistent evidence that individuals with periodontitis have a higher prevalence and incidence of PAD compared to individuals without periodontitis [140]. For cross-sectional data, the most significant evidence comes from two large, population-based studies in the USA (NHANES 1999–2002) and South Korea (KoGES-CAVAS). Both studies found a positive association between the extent of clinical attachment loss (NHANES 1999–2002) and severity of radiographic bone loss (KoGES-CAVAS) with PAD, defined using the Ankle Brachial Index (ABI), with adjusted odd ratios (OR) of 2.2 (95% confidence interval -CI-; [1.2; 2.4]) and 2.0 (95% CI [1.1; 3.9]), respectively [2,70]. One prospective cohort study, conducted in male veterans in the USA, reported a positive association between periodontitis (measured by severity of radiographic bone loss) and the incidence of PAD over a 25–30 year follow-up period, with an adjusted OR of 2.3 (95% CI [1.3; 3.9]) [77]. There are no studies that have evaluated the association between periodontitis and the incidence of Major Adverse Limb Events (MALE).

### 2.5. Do people with periodontitis have a higher risk of other CVDs or conditions (heart failure, atrial fibrillation)?

Several studies report positive associations between periodontitis and heart failure. There is evidence from a large Asian study using the Taiwanese National Health Insurance Research Database reporting a significantly higher incidence of atrial fibrillation in individuals with periodontal diseases compared to individuals without periodontal diseases (hazard ratio –HR = 1.31, 95% CI [1.25; 1.36]) [18].

### 2.6. Do people with a history of cardiovascular disease have a different incidence or progression of periodontitis?

There is currently limited scientific evidence that CVD is a risk factor for the onset or progression of periodontitis.

### 2.7. Do people with periodontitis with history of cardiovascular disease have a higher chance of experiencing a subsequent event?

From three studies investigating the association between periodontitis and secondary cardiovascular events, two large studies did not find a significant association [31,100]; however, a small study (100 subjects) reported a significant association (HR = 2.8, 95% CI [1.2; 6.5]) with recurrent cerebro-vascular events [116].

## 3. Mechanisms that may explain the epidemiological associations between periodontitis and CVD

### 3.1. Is there evidence of a higher incidence of bacteremia following oral function/intervention in periodontitis patients compared to periodontally healthy subjects?

There is evidence that oral bacterial species can enter the circulation and cause bacteremia, which has been demonstrated following daily life activities (tooth brushing, flossing, chewing or biting an apple), although it has been studied more frequently following professional interventions (tooth polishing, scaling, tooth extraction, surgical extraction of third molars and periodontal probing).

The risk of bacteremia has been associated with periodontal health status in a systematic review, suggesting a higher risk of bacteremia associated with gingival inflammation [127]. A recent randomized clinical trial (RCT) concluded that periodontal therapy (by means of scaling and root planing, SRP) induced bacteremia in both gingivitis and periodontitis patients, but the magnitude and frequency were greater among periodontitis patients [5].

Whilst there are methodological limitations in some of the reported studies, the overall picture supports the contention that bacteremia results from daily life activities and oral interventions, and it is more frequent, of longer duration and involves more virulent bacteria in periodontitis patients.

### 3.2. Is there evidence for the presence of oral bacteria in atheroma lesions?

There is evidence through traces of DNA, RNA or antigens derived from oral bacterial species, mainly periodontal pathogens, that have been identified in atherothrombotic tissues. Studies have attempted to correlate the presence of these bacteria in atherothrombotic tissues with other sample sources (subgingival plaque, serum, etc.) in the same patients, and these suggest that in periodontitis patients there is a higher probability of a positive correlation [3,73]. At least two studies have demonstrated viable *P. gingivalis* and *A. actinomycetemcomitans* in athero-thrombotic tissue when culturing the atheroma samples [56,98].

### 3.3. Do we have evidence that periodontal bacteria and/or bacterial products and virulence factors influence the pathophysiology of atherosclerosis?

Different animal models have been employed to provide evidence that periodontal pathogens can promote atheroma formation. *P. gingivalis* has been shown to accelerate atherosclerosis in murine models, to induce fatty streaks in the aorta of rabbits and to induce aortic and coronary lesions after bacteremia in normocholesterolaemic pigs (Schenkein & Loos. 2003).

Recently, further evidence has emerged using hyperlipidemic ApoEnull mice after infection with *P. gingivalis* and also with a polymicrobial experimental infection (*P. gingivalis, Treponema denticola, Tannerella forsythia* and *Fusobacterium nucleatum*). A polymicrobial infection was shown to induce aortic toll-like receptor (TLR) and inflammasome signaling, with an enhanced oxidative stress reaction generated within the aortic endothelial cells [20, 131, 132].

There is also *in vitro* evidence of intracellular entry by periodontal pathogens (*P. gingivalis, A. actinomycetemcomitans*, etc.) [101]. *In vivo* and *in vitro* studies demonstrate the importance of the fimbriae of *P. gingivalis* to host cell entry and to promote atherothrombotic lesions in experimental models [138]. *In vitro* experiments have shown certain bacterial strains expressing *P. gingivalis* hemagglutinin A (HagA) have an increased capability to adhere and enter human coronary artery endothelial cells (HCAEC) [7].

### 3.4. Do we have evidence that periodontitis patients exhibit increased production and/or levels of inflammatory mediators also associated with the pathophysiology of atherosclerosis?

There is evidence of significantly higher levels of C-reactive protein (CRP) in periodontitis patients versus healthy controls and in CVD and periodontitis patients compared with either condition alone. The effect of periodontal therapy has been shown to associate with a significant decrease in CRP levels, along with improvements in surrogate measurements of cardiovascular health [27, 55, 93].

There is evidence of elevated levels of serum interleukin (IL)-6 in periodontitis patients and lower levels of IL-4 and IL-18. The effect of periodontal therapy has shown a significant decrease in the serum levels of IL-6, serum amyloid A and alpha 1 anti-chymotripsin. Peripheral neutrophils from periodontitis patients release excess IL-1β, IL-8, IL-6 and tumour necrosis factor (TNF)-α when stimulated by periodontal pathogens. Periodontal therapy only partially reduces the cytokine hyperreactivity with some evidence of a constitutively elevated response [66].

### 3.5. Do we have evidence that periodontitis patients develop elevations in thrombotic factors that are also associated with the pathophysiology of atherothrombosis?

There is evidence of significantly higher levels of fibrinogen in periodontitis patients versus healthy controls and in CVD and periodontitis patients compared with either condition alone [15]. Periodontal therapy appears to result in a significant decrease in fibrinogen levels [69,133].

There is evidence from different studies of significantly higher levels of platelet activation markers in periodontitis patients and that these higher levels may be reversed by periodontal therapy [4]. However, there is conflicting evidence that significantly higher levels of plasminogen activator inhibitor (PAI) are found in periodontitis patients (Schenkein & Loos. 2003).

### 3.6. Do we have evidence that periodontitis patients demonstrate elevated serum antibody levels that cross-react with antigens in cardiovascular tissues?

There is evidence that Heat Shock Proteins (HSP) from periodontal pathogens (*Porphyromona gingivalis, Tannerella forsythia, Aggregatibacter actinomycetemcomitans* and *Fusobacterium nucleatum*) generate antibodies that can cross-react with human HSPs. These antibodies have been shown to activate cytokine production, as well as monocyte and endothelial cell activation.

Presence of anti-cardiolipin antibodies have been significantly associated in periodontitis patients, which reversed following periodontal therapy. There is some evidence that periodontal pathogens can elicit antibodies that cross-react with cardiolipin (Schenkein & Loos. 2003).

In three out of four population-based studies (Parogene study, NHANES III, DANHES), higher levels of serum immunoglobulin (Ig)G against *P. gingivalis* were associated with periodontitis patients and cardiovascular disease (acute coronary syndrome, death from cardiovascular disease and cardiovascular disease). The ATHEROREMO-IVUS study failed to demonstrate an association between serum levels of IgG and IgA against *P. gingivalis, A. actinomycetemcomitans, T. forsythia* and *P. intermedia* and major adverse cardiac events (MACE) [24]. This is consistent with data from Boillot et al. [11].

### 3.7. Do we have evidence that periodontitis patients exhibit dyslipidemia?

There is evidence from systematic reviews that serum total cholesterol levels, low-density lipoproteins (LDL), triglycerides, very low-density lipoproteins (VLDL), oxidized LDL and phospholipase A2 are elevated in periodontitis. High-density lipoprotein (HDL) levels are reduced in periodontitis patients compared with controls (Schenkein & Loos. 2003). These levels are reversed after periodontal therapy [125].

### 3.8. Do we have evidence for peripheral blood neutrophil hyperresponsiveness in reactive oxygen species and protease production in periodontitis patients?

There is strong mechanistic evidence that peripheral blood neutrophils (PBNs) from periodontitis patients produce higher levels of total and extracellular reactive oxygen species (ROS) than healthy controls, under various conditions of priming and stimulation and from unstimulated cells [66,74]. This hyper-reactivity to stimulation by periodontal bacteria is reduced following successful periodontal therapy to control patient levels, but the unstimulated hyperactivity remains, suggesting constitutive and reactive mechanisms underlie neutrophil hyper-responsiveness in periodontitis [75]. Gene expression data in PBNs supports the functional data [136]. Serum antioxidant levels and those in gingival crevicular fluid (GCF) are reduced in periodontitis patients, reflecting increased ROS activity [16]. This data is supported by a study of endarterectomy samples, which demonstrated evidence for activation of the ROS-generating systems in neutrophils, specifically the presence of myeloperoxidase (MPO), cell-free DNA and DNA-MPO complexes [99].

### 3.9. Are there common genetic risk factors between periodontitis and CVDs?

There is scientific evidence of pleiotropy between periodontitis and cardiovascular diseases [1, 83, 111, 112]. The highly pleiotropic genetic locus *CDKN2B-AS1* (chromosome 9, p21.3) associated with coronary artery disease, type 2 diabetes, ischemic stroke and Alzheimer’s disease is also consistently associated with periodontitis [1, 33, 68, 83]. Its function appears to be related to the regulation of gene expression [48]. Interestingly, a pilot study identified that a genetic variant in the *CDKN2B-AS1* locus was associated with the extent of elevated levels of C-reactive protein in periodontitis [124].

A conserved non-coding element within *CAMTA1* upstream of *VAMP3*, also first identified as a genetic susceptibility locus for coronary artery disease, was found to be associated with periodontitis [111]. A GWAS suggested the *VAMP3* locus was associated with a higher probability of subgingival overgrowth of periodontal pathogens [29].

There is evidence for plasminogen (PLG) as a shared genetic risk factor for coronary artery disease and periodontitis [111].

The fourth pleiotropic locus between coronary artery disease and periodontitis is a haplotype block at the *VAMP8* locus [83].

These shared genetic factors suggest a mechanistic link or immunological commonalities between coronary artery disease and periodontitis. The impairment of the regulatory pathways by genetic factors may be a common pathogenic denominator of at least coronary artery disease and periodontitis. There are indications that aberrant inflammatory reactivity, determined by genetic variants in the loci *CDKN2B-AS1 (ANRIL), PLG, CAMTA1/VAMP3* and *VAMP8* could partially explain the epidemiological link between periodontitis and cardiovascular diseases.

## 4. Evidence from intervention studies

### 4.1. Is there an effect of periodontitis treatment in preventing or delaying ACVD events?

#### 4.1.1. Primary Prevention

There have been no prospective randomized controlled periodontal intervention studies on primary prevention of cardiovascular diseases (including first ischemic events or cardiovascular death) since the last consensus report [128]. The Group questioned the feasibility of performing adequately powered RCTs in primary prevention at a population level due to important ethical, methodological and financial considerations.

However, consistent observational evidence suggests several oral health interventions, including self-performed oral hygiene habits (toothbrushing) (two studies: [25,91]), dental prophylaxis (one study: [60]), increased self-reported dental visits (one study: [115]) and periodontal treatment (three studies: [47, 60, 91] produced a reduction in the incidence of ACVD events.

Cross-sectional data of the Scottish Health Surveys from 1995 to 2003 pertaining to 11,869 men and women (mean age of 50 years) was linked to a database of hospital admissions and deaths with follow-up until December 2007 (Information Services Division, Edinburgh) [25]. Participants who brushed less than once a day exhibited the highest incidence of ACVD events (HR = 1.7, 95% CI [1.3; 2.3]) compared with those who brushed twice a day, indicating that self-performed oral hygiene routines may reduce the incidence of ACVD.

A retrospective nationwide, population-based study in Taiwan, including 511,630 participants with periodontitis and 208,713 controls, used the Longitudinal Health Insurance Database 2000 to estimate the incidence rate of ACVD events from 2000 to 2015 [60]. The hazard ratio for acute myocardial infarction was reduced more in the group of periodontitis patients who received dental prophylaxis (HR = 0.90, 95% CI [0.86; 0.95]) than intensive treatment (including gingival curettage, scaling and root planing, and/or periodontal flap operation and/or tooth extraction) (HR = 1.09, 95% CI [1.03; 1.15]). Consistent reductions in the incidence rate of Ischemic Stroke were observed in both the dental prophylaxis (HR = 0.78, 95% CI [0.75; 0.91]) and intensive treatment groups (HR = 0.95, 95% CI [0.91; 0.99]).

A cohort of 8,999 patients with periodontitis who received a complete (non-surgical and if needed surgical) periodontal treatment protocol were followed between 1979 and 2012 [47]. During the study follow-up, poor responders to the periodontal treatment had an increased incidence of ACVD events (incidence rate –IR = 1.28, 95% CI [1.07; 1.53]) compared with good responders, suggesting successful periodontal treatment could reduce the incidence of ACVD events.

In the Atherosclerosis Risk in Communities (ARIC) study, including 6,736 participants followed during 15 years, self-reported regular dental care users had a lower risk for ischemic stroke (HR = 0.77, 95% CI [0.63; 0.94]) compared to episodic care users [115].

A prospective population-based study using data from the National Health Insurance System-National Health Screening Cohort (NHISHEALS), including 247,696 participants free from any CVD history recruited between 2002 and 2003, reported an increased number of dental caries lesions, the presence of periodontitis and a greater loss of teeth were all associated with an increased risk of future major cardiovascular events (MACEs), including cardiovascular death, acute myocardial infarction, heart failure and stroke [91]. One additional toothbrushing episode per day was associated with a reduced incidence of ACVD events (HR = 0.91, 95% CI [0.89, 0.93]), and regular professional cleaning reduced the risk even further (HR = 0.86, 95% CI [0.82; 0.90]).

In summary, progression of ACVD may be influenced by successful periodontal treatment independent of traditional CVD risk factor management.

#### 4.1.2. Secondary Prevention

There is only one pilot multi-centre study on secondary prevention of ACVD events (PAVE: [21,88]), which reported no statistically significant difference in the rate of CVD events between patients who underwent treatment of periodontitis versus community care (risk ratio –RR = 0.72, 95% CI [0.23; 2.22]). Several methodological limitations highlighted in the trial limit the applicability/usefulness of such evidence to inform the research and healthcare communities.

Thus, there is insufficient evidence to support or refute the potential benefit of the treatment of periodontitis in preventing or delaying ACVD events [61].

### 4.2. What is the effect of the treatment of periodontitis in improving surrogate parameters of CVD?

Table 1 summarizes the evidence on the effect of periodontal therapy on surrogate markers of CVD. There is moderate evidence for reduction of low-grade inflammation as assessed by serum levels of CRP, IL-6 and improvements in surrogate measures of endothelial function (flow-mediated dilatation of the brachial artery).

**Table 1:** Summary of the evidence on the effect of periodontal therapy on surrogate markers of cardiovascular diseases.

Topic	Outcome	Number of RCTs and SR since last consensus	References	Effect	Overall Level of Evidence

**Effect of Periodontal Therapy on Lipids**	Lipids (multiple)	6 RCTs	[12, 22, 26, 38, 44, 51]	No	Moderate
**Effect of Periodontal Therapy on Blood Pressure**	Systolic, diastolic	3 RCTs	[22, 44, 142]	Yes	Limited
**Effect of Periodontal Therapy on Endothelial Function**	Endothelial Function (multiple measures)	2 RCTs	[22, 105]	Yes	Moderate
		1 SR	[122]		
**Effect of Periodontal Therapy on interleukin (IL)-6**	IL-6	3 RCTs	[38, 51, 142]	Yes	Moderate
**Effect of Periodontal Therapy on C-Reactive Protein (CRP)**	CRP	5 SR	[27, 37, 49, 90, 125]	Yes	Moderate
		7 RCTs following 2014	[12, 22, 26, 44, 51, 53, 142]		
**Effect of Periodontal Therapy on Pulse Wave Velocity (PWV)**	PWV	1 RCT	[51]	No	Limited
**Effect of Periodontal Therapy on carotid Intima-Media Thickness (c-IMT)**	Common c-IMT	1 RCT	[51]	Yes	Limited

RCT, randomised clinical trial; SR, systematic review.

Moderate evidence suggests periodontal treatment does not have an effect on lipid fractions, whilst there is limited evidence suggesting periodontal treatment reduces arterial blood pressure and stiffness, subclinical ACVD (as assessed by mean carotid Intima-Media-Thickness), and there is insufficient evidence of an effect on ACVD biomarkers of coagulation, endothelial cell activation and oxidative stress.

### 4.3. What is the effect of statin intake on clinical periodontal outcomes?

Statins are medications prescribed to decrease LDL cholesterol. Numerous trials have demonstrated their benefit for the prevention of cardiovascular diseases [141].

Interestingly, statins possess various additional properties relevant to the pathogenesis and treatment of periodontitis [34]. In particular, it has been reported that statins are anti-inflammatory [54, 94, 97, 102, 106], can promote bone formation [41, 67, 81, 134], can inhibit matrix metalloproteinases (MMPs) [54, 71, 96] and possess anti-microbial properties [126].

A systematic review with meta-analysis of pre-clinical in vivo trials reported a positive effect of local or systemic statin administration for the prevention of alveolar bone loss in experimental periodontitis models in rodents [9].

Several observational clinical studies have evaluated the effect of systemic statin intake on periodontal conditions [65, 76, 107, 109, 110, 123]. Statin use was not found to be associated with decreased tooth loss in adults with chronic periodontitis when analysing administrative health plan data [109]. However, a five-year population-based follow-up study comparing participants treated with statins with those who did not medicate with statins concluded long-term treatment with statins was associated with reduced tooth loss [76]. Furthermore, patients on statin medication were reported to exhibit significantly fewer signs of periodontal inflammatory lesions than patients without a statin regimen [65]. A cross-sectional study compared the periodontal status of patients with hyperlipidaemia (with or without statin intake) to normolipidaemic individuals and found higher gingival bleeding and probing depths in the hyperlipidaemic patients who were not statin users [107]. In an RCT, periodontal patients with risk factors or with established atherosclerosis were assigned to either a high or low dose statin intake [123]. After three months, a significant reduction of periodontal inflammation was seen in the high-dose compared to the low-dose group. Thus, within the limits of the above reported studies, there is some limited evidence suggesting statins may have a positive impact on periodontal health.

Very few clinical studies have been designed to evaluate the effect of adjunctive systemic statin intake in conjunction with periodontal therapy [35, 36, 108]. In a randomized placebo-controlled pilot study in 38 patients with chronic periodontitis, adjunctive statin intake led to beneficial effects on radiological bone loss and tooth mobility after 3 months [35]. Another 3 month-study compared the treatment response to nonsurgical periodontal therapy in 107 chronic periodontitis patients (35 normolipidemic as control, 36 hyperlipidaemic on nonpharmacological therapy and 36 hyperlipidemic on statins) and found a greater improvement in gingival index in the nomolipidemic control and in the statin groups [108]. Based on this limited evidence, two recent systematic reviews with meta-analysis on the effects of (local and systemic) statins on periodontal treatment concluded systemic statin intake does not enhance the outcomes of periodontal therapy [8,82].

## 5. Cardiovascular risks and complications of periodontal therapeutic interventions

### 5.1. Is there an ischemic cardiovascular risk for patients undergoing periodontal therapy?

Non-surgical treatment of periodontitis involving supra- and sub-gingival instrumentation of the affected dentition (under local anaesthesia) is often delivered in several short sessions. Alternatively, full-mouth non-surgical periodontal treatment can be performed within 24 hours.

Delivering periodontal treatment in a full-mouth fashion (i.e., within 24 hours) triggers a one-week acute systemic inflammatory response associated with a transient impairment of endothelial function [89]. This distant effect is not observed when periodontal treatment is delivered across several separate sessions [42]. This is achieved by limiting the number of teeth involved and the time devoted to completing the dental instrumentation. These findings raise the question of whether performing longer sessions of periodontal treatment could contribute to an individual’s inflammatory burden/risk and increase their short-term risk of suffering from a vascular event. There is consistent and strong observational evidence that common acute infections/inflammatory responses are associated at a population level with an increased risk of vascular events within the first four weeks of the infectious/inflammatory event [120].

#### 5.1.1. At population level

There is no evidence for specific effects of periodontal treatment procedures on increasing ischemic cardiovascular risk. Two observational studies reported no effect of *invasive dental treatment* in elevating ischemic cardiovascular risk [19,86], and one study suggested a minimal increased risk within four weeks following treatment [79].

Chen et al. [19] performed a case-crossover and self-controlled case series using the Taiwanese National Health Insurance Research Database, including over 110,000 myocardial Infarction cases and 290,000 Ischemic Stroke patients, between 1999 and 2014. They reported a non-significant increase in the incidence of myocardial infarction within the first 24 weeks following *invasive dental treatment* (including periodontal procedures) except for a modest risk of myocardial infarction during the first week for patients without other comorbidities (OR = 1.31, 95% CI [1.08; 1.58], after 3 days).

A registry-based case-control study between 2011 and 2013 including 51,880 cases who underwent an *invasive dental procedure* compared to 246,978 controls reported no association with increased incidence of myocardial infarction (OR 0.98, 95% CI [0.91; 1.06]) [86].

Minassian et al. [79] performed a self-controlled case series including nearly 10 million participants included in an insurance database from 2002 and 2006 in the USA. The analysis showed that *invasive dental treatment* (largely comprising of tooth extractions and only 4% being non-surgical and surgical periodontal procedures) is associated with an increased risk of incident acute cardiovascular events (IR = 1.5, 95% CI [1.09; 2.06]) within the first four weeks of treatment recorded.

In summary, the Group concluded delivering periodontal treatment is safe with regard to cardiovascular risk.

#### 5.1.2. In patients with established CVD

There is limited evidence on the effects of *invasive dental treatment* on the incidence of Ischemic Events in patients with established CVD or after an event.

A small RCT on the effects of the treatment of periodontitis on CVD biomarkers in patients with established CVD [80] showed no cardiovascular adverse events within three months of completion of scaling and root planing (periodontal therapy).

In the PAVE feasibility randomized secondary prevention trial, provision of periodontal scaling and root planing treatment in patients with established CVD did not increase the incidence of cardiovascular events compared to the control group (community treatment) within six months [6].

In summary, the Group concluded delivering periodontal treatment is safe with regards to cardiovascular risk in patients with established CVD.

### 5.2. What is the perioperative bleeding risk when performing periodontal therapy?

Periodontal treatment consists of numerous procedures with different levels of bleeding risk. However, this risk of bleeding is low in the vast majority of procedures and can be easily controlled with local haemostatic measures.

Perioperative bleeding risk varies according to the extent and invasiveness of the periodontal procedure performed. The majority of periodontal procedures may be grouped within the ESC/AHA/EHRA [121,122]. Low Bleeding Risk (frequency less than 1% of post-operative bleeding) group: supragingival polishing, non-surgical periodontal treatment, conventional surgical periodontal treatment (conservative, resective or regenerative), tooth extractions and dental implant placement. Moderate bleeding risk (frequency between 2–5%) may be observed in major autogenous bone augmentation procedures, such as block bone harvesting, sinus floor elevation and procedures where healing is by secondary intention, such as free gingival grafting. Appendix 1 summarizes the main recommendations for patients with antithrombotic therapy when perfoming periodontal therapy.

#### 5.2.1. In patients undergoing anti-platelet therapy

Individuals undergoing single acetylsalicylic acid (ASA) therapy (aspirin) in different therapeutic dosages, as well as therapy with clopidogrel, ticlopidine or ticagrelor show no statistically significant differences in frequency of bleeding events when compared to controls (i.e., subjects not undergoing anti-platelet therapy) [30,62].

Dual antiplatelet therapy, most commonly ASA in combination with clopidogrel, may pose a certain risk for postoperative bleeding complications; however, it appears these haemorrhagic events may be managed safely with local haemostatic measures [84,85].

Thus, current evidence does not support discontinuation of antiplatelet therapy before dental procedures, irrespective of the type of therapy employed (single or dual antiplatelet therapy) or the type of procedure performed (single, multiple tooth extractions, non-surgical and surgical periodontal therapy and dental implant procedures).

#### 5.2.2. In patients undergoing anti-coagulant therapy

##### 5.2.2.1. Vitamin-K antagonists

In patients taking oral anticoagulant therapy (vitamin-K antagonists, VKA) and undergoing dental extraction, minor dental procedures and dental implant placement do not seem to increase the risk of bleeding compared to patients who discontinue oral anticoagulant therapy [118,139]. There may be a higher post-operative bleeding risk in patients continuing VKA and undergoing either minor dental surgery or other higher-risk procedures when compared to non-VKA patients [10,118], but local haemostatic agents appear to be effective in controlling post-operative bleeding [72].

##### 5.2.2.2. Novel/direct anticoagulants (DOAC/NOAC)

Limited trials and evidence are available on the management of patients on novel oral anticoagulant (NOAC) therapy undergoing dental treatment; hence, the Group concluded further studies regarding dental procedures in these patients are strongly encouraged.

It appears there is no need for interruption of NOAC therapy in most dental treatments due to a low incidence of bleeding complications, which can be successfully managed with local haemostatic measures when comparing groups continuing NOAC and groups discontinuing NOAC therapy [58, 59, 92, 137], and with reported timing of discontinuation and reinstitution varying greatly. When comparing NOAC patients with healthy individuals, there seems to be a higher incidence of delayed bleeding (2 days and later) in those patients who do not discontinue NOAC therapy [78].

## 6. Recommendations

### 6.1. Recommendations for oral health professionals for use in dental practice/office for people with cardiovascular disease (CVD)

- Patients with periodontitis should be advised there is a higher risk for cardiovascular diseases, such as myocardial infarction or stroke, and as such they should actively manage all their cardiovascular risk factors (smoking, exercise, excess weight, blood pressure, lipid and glucose management and sufficient periodontal therapy and periodontal maintenance).
- Patients with periodontitis and a diagnosis of CVD should be informed they may be at higher risk for subsequent CVD complications and, therefore, they should regularly adhere to the recommended dental therapeutic, maintenance and preventive regimes.
- Collect a careful history to assess for CVD risk factors, such as diabetes, obesity, smoking, hypertension, hyperlipidemia and hyperglycemia. Suggestthe patient consults his/her physician if any of these risk factors are not appropriately controlled.
- Oral health education should be provided to all patients with periodontitis and a tailored oral hygiene regime, including twice-daily brushing, inter-dental cleaning and in some cases the use of adjunctive chemical plaque control may be appropriate.
- People presenting with a diagnosis of CVD should receive a thorough oral examination, which embeds a comprehensive periodontal evaluation, including full-mouth probing and bleeding scores.
- If no periodontitis is diagnosed initially, patients with CVD should be placed on a preventive care regime and monitored regularly (at least once a year) for changes in periodontal status.
- In people with CVD, if periodontitis is diagnosed, they should be managed as soon as their cardiovascular status permits.
- Irrespective of the level of CVD or specific medication, non-surgical periodontal therapy should be provided, preferably in several 30–45 minute sessions to minimize a spike of acute systemic inflammation
- Surgical periodontal and implant therapy, when indicated, should be provided in a similar manner as in patients without CVD.

However, attention should be paid to:

- Hypertension. It is recommended to measure the patients’ blood pressure (after appropriate relaxation) before the surgical intervention, and in cases of high blood pressure (above 180/100 according to expert opinion), the surgery should be postponed until the patient’s blood pressure is stabilized.
- Medication with antiplatelet and anticoagulant drugs. Because periodontal and implant surgical procedures usually impart only a low to medium risk of bleeding in general terms, the dentist should not change a patient’s medication or in cases of doubt he/she should consult the physician/cardiologist prior to the surgical intervention. Consideration should also be given to the local management of bleeding complications that may arise.

Current AHA/ACC/SCAI/ACS/ADA/ESC/ACCP guidelines on perioperative management of antithrombotic therapy do not suggest discontinuation of anti-platelet therapy for Low Bleeding Risk procedures [32, 43, 57].

Various approaches for perioperative management of anticoagulant therapy have been suggested. The Group reviewed the guidelines on perioperative management of vitamin K antagonists (VKA) and suggests discontinuation of medication treatment if the INR is 4 or below for Low or Medium Bleeding Risk procedures [95]. However, if the internationalised normalised ratio (INR) is 3.5 or above, the expert Group recommends dental clinicians seek advice and consult with the responsible medical professional. Management of high thromboembolic risk cases should be collaborative in consultation with the medical professional responsible for the VKA therapy [57,130].

After reviewing novel anticoagulant (non VKA) and direct anticoagulant (NOAC/DOAC) therapies guidelines, the Group concluded that for Low Bleeding Risk periodontal procedures no discontinuation of anticoagulants is recommended [121,122]. These procedures could be performed 18–24 hrs after the last intake (depending on a renal function assessment for the medication in question) and then restarted 6 hrs following treatment. The expert Group, however, strongly recommends the dental clinician consult with the responsible medical professional. When a Medium Bleeding Risk periodontal procedure is planned, discontinuation of therapy should be agreed with the medical professional responsible for and/or prescribing the anticoagulant therapy.

Lastly, in cases of combined anti-platelet and anticoagulant therapies, which pertain to patients with the highest thrombotic and ischemic risk (i.e., chronic atrial fibrillation or after an acute myocardial infarction or recent coronary stenting), when periodontal procedures (either of Low or Medium Bleeding Risk) are required, any alterations in medication should be discussed and agreed upon with the responsible medical professional [121,122]. In elective periodontal procedures, the operation should be delayed until after treatment stabilization and appropriate consultation with the medical specialist.

In cases of triple therapy (dual anti-platelet and one anticoagulant) or one anticoagulant plus one anti-platelet, such patients need individualised management by the responsible medical professional according to their thrombotic and haemorrhagic risk [130].

It is important to highlight that local haemostatic agents (such as oxidised cellulose, absorbable gelatin sponges, sutures, tranexamic acid mouthwashes, compressive gauze soaked in tranexamic acid) should be used and dental clinicians should consider the confounding effect of local anaesthetic with vasoconstrictors.

- Patients with a risk of endocarditis should be premedicated with antibiotics following current guidelines (such as the European or the American guidelines).
- People with cardiovascular disease who have extensive tooth loss should be encouraged to pursue dental rehabilitation to restore adequate mastication for proper nutrition.
- People without a diagnosis of CVD but with risk factors for CVD should be informed about their CVD risk and referred to a physician for appropriate risk assessment, diagnostic testing and follow-up care. For oral health professionals, risk assessment may be performed based upon the recommendations of the *European Society of Cardiology (Systematic COronary Risk Evaluation, SCORE)* [119].

### 6.2. Recommendations for physicians and other medical health professions for use in cardiology practice

Because of the potential negative impact of periodontitis on CVD complications, the following recommendations are made:

- Patients with CVD should be advised periodontitis may have a negative impact on CVD and may also increase the risk of CVD events.
- Patients should be advised effective periodontal therapy may have a positive impact upon CV
- health.
- For people with CVD, physicians should ask about a prior diagnosis of periodontitis. If a positive diagnosis has been made, the physician should seek to ascertain that appropriate periodontal care and maintenance are being provided.
- Patients with CVD should be asked about any signs and symptoms of periodontitis, including bleeding gums during brushing or eating, loose teeth, spacing or spreading/drifting of the teeth, oral malodor and/or abscesses of the gums or gingival suppuration.
  - If a positive history is elicited, then a prompt periodontal evaluation should be recommended before their scheduled annual check-up.
  - In the case of a negative history, people with CVD should be advised to check for the above symptoms, and if a positive sign appears, they should visit their dentist at least once per year.
- For all patients with newly diagnosed CVD, referral for a periodontal examination should occur as part of their ongoing management of CVD. Even if no periodontitis is diagnosed initially, an annual oral/dental check-up is recommended.
- The physician should liaise with the dental surgeon over periodontitis management in CVD patients on anticoagulant/antiplatelet therapy prior to the oral intervention and/or periodontal surgery to avoid excess bleeding or the risk of ischaemic events.

### 6.3. Recommendations for patients at the dental surgery/office who have CVD or are found to be at risk of CVD

- People with CVD must be aware that gum disease is a chronic condition, which may aggravate their CVD, and requires lifelong attention and professional care.
- There is a need to clean the teeth and gums very carefully at home. Personalized advice will be provided by the oral health professional.

This may include

- Twice-daily brushing with either a manual or electric toothbrush;
- Cleaning between teeth using inter-dental brushes where they fit; where they do not fit, then flossing may be useful;
- Use of specific dentifrices and/or mouth rinses with proven activity against dental plaque, if advised by oral health professionals;
- If left untreated, gum disease can lead to tooth loss and may also make CVD preventive measures harder to control;
- Gum disease may be present and deteriorate with no apparent symptoms, so the dentist should advise their patient that even without current gum disease, they should still receive regular dental check-ups as part of managing their CVD.

Dentists should be able to identify the early signs of gum disease, but patients should also suspect gum disease if noticing

- Red or swollen gums;
- Bleeding from the gums or blood in the sink after tooth brushing;
- Foul taste;
- Longer looking teeth;
- Loose teeth;
- Increasing spaces between teeth/teeth moving apart;
- Calculus (tartar) on teeth.

Patients should inform their dentist about the outcome of their visits to the physician and provide an update on their CVD history and any changes in medications. Patients should inform the dentist if they are on anticoagulant therapy.

Patients should understand it is important to keep their mouth and whole body as healthy as possible with regular dental and medical visits.

### 6.4. Recommendations for patients with CVD at the physician’s practice/office

#### 6.4.1. Why should I have my gums checked?

If your physician has told you that you have cardiovascular disease (CVD), you should make an appointment with a dental surgeon to have your mouth and gums checked.

This is because people with CVD may have a higher chance of getting further complications when they have gum disease. The earlier you seek help, the better the outcome will be.

#### 6.4.2. What should I look for that may tell me I have problems with my gums?

You may have gum disease if you have ever noticed

- Red or swollen gums;
- Bleeding from your gums or blood in the sink after you brush your teeth;
- Foul taste;
- Longer looking teeth;
- Loose teeth;
- Increasing spaces between your teeth, or your teeth drifting apart;
- Calculus (tartar) on your teeth.

If you have noticed any of these problems, it is important to see a dentist as soon as possible.

#### 6.4.3. Can I have gum disease without these signs being present?

Gum disease may also be present and get worse with no apparent that you have it, especially if you smoke, so even if you do not think you have gum disease now, you should still have annual check-ups of your mouth as part of managing your CVD. Your dentist will be able to pick up early signs of gum disease.

#### 6.4.4. What can I do to prevent gum disease?

You need to clean your teeth and gums twice daily at home for a minimum of two minutes. Also, cleaning between your teeth daily is important, and your oral health professional will show you how to do this. You should visit a dental surgeon as soon as possible for a diagnosis and advice on what you need to do. It is important to keep your mouth as healthy as possible with regular oral and dental care, according to the recommendations of your oral health professional.

## Additional File

The additional file for this article can be found as follows:

10.5334/gh.400.s1 Supplementary Appendix 1. Antithrombotic therapy: when, how and why. Comprehensive approach for oral health professionals.
