# Supplementary material for: Periodontitis and Cardiovascular Diseases. Consensus Report
Source: Glob Heart. 2020 Feb 3;15(1):1. doi: 10.5334/gh.400 (PMC7218770; doi:10.5334/gh.400)
Supplement: Supplementary Appendix 1. — Antithrombotic therapy: when, how and why. Comprehensive approach for oral health professionals. [file gh-15-1-400-s1.pdf]

Supplementary Appendix 1. Antithrombotic therapy: when, how and why. Comprehensive approach for oral health professionals.

1. Introduction

The use of antithrombotic therapy is one of the cornerstones of cardiovascular medicine, as the main pathophysiological event is thrombus formation. The widespread use of antithrombotic agents is an indisputable fact and is increasing everyday with the ageing of the general population and the unstoppable growth of cardiovascular diseases (CVD) prevalence. Altogether, the number of available agents, indications and timing of these therapeutic interventions is a matter of constant discussion and controversy. This document pretends to complement the current recommendations of the Joint Committee of the European Federation for Periodontology (EFP) and the World Heart Federation (WHF) with an overview of the rationale of the antiplatelet and anticoagulant therapies in the setting of cardiovascular disease, to increase awareness of the dos and don´ts of these medications and optimize the thrombotic and bleeding of patients with CVD that undergo periodontal interventions.

2. Pharmacology of antithrombotic agents

Thrombus prevention is based on the interruption of the haemostasis, and this can be achieved by intervening in the primary haemostasis (namely, platelet function) or secondary haemostasis (basically, humoral factors). Agents whose main target is primary haemostasis are usually called antiplatelet drugs and are widely used in circumstances in which the main phenomenon is local thrombosis (e.g. myocardial infarction, non-embolic strokes, etc.). On the other hand, drugs that alter secondary haemostasis are generally referred to as anticoagulants and are used in conditions that increase the risk of clot formation with subsequent embolization (e.g. atrial fibrillation, deep vein thrombosis, etc.).

Antiplatelet drugs

The currently commercialized antiplatelet drugs of common use are:

- Acetylsalicylic acid (ASA - Adiro®)
- AntiP2Y12: usually, these medications are used as adjuvants in situations of abnormally high thrombotic risk, such as the months after a myocardial infarction (MI) or the placement of a coronary stent. When compared with ASA, all of these drugs have a higher antithrombotic power and, thus, a higher bleeding risk. That is why it is of no surprise that, when used as adjuvants therapies together with ASA, the final risk of bleeding arises from a synergistic

effect of both drugs and is markedly elevated compared with single antiplatelet therapy. As the thrombotic risk in the aforementioned situations (recent MI, recent coronary stent placement) decreases with time, the use of these agents is almost always limited for a number of months after the event. These group includes:

- Clopidogrel (Plavix®)
- Ticagrelor (Brilique®, Brilinta®, Ticalog®)
- Prasugrel (Efient®, Effient®, Agrepres®, Prasugil®, Prasita®)

The last two agents, namely ticagrelor and prasugrel, were released several years after the commercialization of clopidogrel due to some concerns regarding the efficacy of the latter in selected populations with resistance its biological effect. Both medications have shown a higher platelet inhibition when compared to clopidogrel with a logically expected increase in the bleeding rates. No studies have addressed the comparative risk of ticagrelor against prasugrel.

There are other available antiplatelet drugs, but their use is restricted to very specific and rarely situations that are beyond of the scope of this review and, thus, would not be review.

Anticoagulant drugs

The span of anticoagulant drugs is broader, as it included both oral and parenteral families. The oral anticoagulant medications include:

- Vitamin K antagonists (VKA): These anticoagulants were the first oral agents in the market and have been available for more than 20 years. Their use is widely extended, as they are very cheap agents and the medical community is very comfortable with its use. Another positive feature is that the dose can be titrated to achieve higher or lower anticoagulant effect, depending on the thrombotic risk of the patient. However, they have some setbacks that are to be considered. Firstly, the dose is not predictable, so the patient must routinely undergo haemostasis checks at least monthly to adjust the dosage regime. In addition, these agents are tightly bound to plasmatic proteins and tend to interact with medications that displace them from that union (e.g. non-steroidal anti-inflammatory drugs, antibiotics, etc.). Also, their effect varies widely with vitamin K intake with the diet. All in all, these medications can be quite uncomfortable for both the patient and the doctor: the patient has to be disciplined with the diet and the dosage regime, and should be aware of all the

medications that can interact. Physicians, on the other hand, have to check these patients up monthly to adjust the dose. The active principles of this group are:

- Warfarin (Coumadin®, Farin®, Aldocumar®)
- Acenocumarol (Sintrom®)

While in North America the use of warfarin is much more frequent, acenocumarol is the preferred choice in Europe.

- Direct Oral Anticoagulants (DOACs): this group encompasses four different drugs divided into direct thrombin inhibitors and Xa factor inhibitors. The benefits these medications have in comparison with VKA is that their dose is predictable, so no dose monitoring is necessary. Also, their interactions are limited and much more infrequent. Regarding outcomes, these drugs have shown to be at least non-inferior to acenocumarol in embolic prevention with a better safety profile as measured by a lower bleeding risk. This better overall profile is the reason why DOACs are now the first line therapy with patients with atrial fibrillation (AF) according to the current clinical practice guidelines. However, not all the AF patients are candidates for these medications. This group includes:
    - Dabigatran (Pradaxa®): available as 110 mg or 150 mg tablets. It is used twice a day.
    - Rivaroxaban (Xarelto®): available as 15 mg or 20 mg tablets. It is used once daily.
    - Apixaban (Eliquis®): available as 2.5 mg or 5 mg tablets. It is used twice a day.
    - Edoxaban (Lixiana®): available as 30 mg or 60 mg tablets. It is used once daily.

Other than the above, sometimes patients can be on parenteral anticoagulant therapies such as low molecular weight heparins. These medications are normally used during short periods of time while bridging in between drugs.

3. Indications

For single antiplatelet therapy (SAPT)

Every patient with any form of CVD that belong to the atherosclerotic spectrum (coronary artery disease, cerebrovascular disease or peripheral artery disease) should start indefinite treatment with an antiplatelet agent. For this indication, the most extended practice is to use ASA, but also clopidogrel can be used. Ticagrelor and prasugrel are never used in monotherapy (Neumann et al., 2019; Task Force et al., 2013).

Although this has been widely discussed during several decades, the use of ASA in primary prevention is currently not justified and should be avoided.

Other than the above, there are several conditions that also require the utilization of ASA, such as some cases of antiphospholipid syndrome or the presence of intracardiac structural devices (mitral clip, atrial and/or ventricular septal defect occluders, bioprosthetic valves, etc.)

For double antiplatelet therapy (DAPT)

- Coronary artery disease (CAD): patients with CAD require DAPT in the following situations (Ibanez et al., 2018; Neumann et al., 2019):
  - Chronic angina pectoris: after the implantation of a coronary stent, it is generally recommended to use DAPT for 6 months, after which the patient can discontinue the second antiplatelet drug and stay only on ASA. In some cases of high risk of bleeding, the duration can be shortened to three months. The only combination approved for this scenario is ASA + clopidogrel.
  - Acute coronary syndrome (myocardial infarction): after a myocardial infarction, is it recommended to use DAPT for a duration of 12 months. However, in some cases, the duration can be shortened to only one month, after which the patient should discontinue the second agent and remain on ASA indefinitely. First month after stent implantation is particularly critical, so no patient should suspend DAPT within this time window. Preferably, the patient should be on ASA + ticagrelor/prasugrel, but ASA + clopidogrel is also acceptable if there are contraindications for the other agents.
- Cerebrovascular disease (CeVD): patients with CeVD require DAPT in the following situations:
  - Chronic carotid disease: in symptomatic patients with carotid stenosis that undergo carotid stenting, ASA + clopidogrel is recommended for

one month. After that, the patient can discontinue one of the two agents and remain indefinitely on the other (usually clopidogrel is discontinued).
    - Acute ischemic stroke: the use of DAPT for secondary prevention of stroke has not shown consistent positive results in this scenario. Thus, it is not routinely recommended.
- Peripheral artery disease (PAD) (European Stroke et al., 2011):
    - Chronic lower extremity artery disease: in symptomatic patients that undergo lower limb percutaneous revascularization (stenting), ASA + clopidogrel is recommended for one month. After that, the patient can discontinue one of the two agents and remain indefinitely on the other (usually clopidogrel is discontinued).
    - Acute ischemic lower limb event: the use of DAPT for secondary prevention of lower limb occlusions has not shown consistent positive results in this scenario. Thus, it is not routinely recommended.

Chronic Oral Anticoagulation (COA)

There are three main indications for anticoagulation:

- Deep vein thrombosis (DVT) and pulmonary embolism (PE) (Konstantinides et al., 2014): both VKA and DOACs are authorized for this indication. The duration of the therapy depends on whether it is assumed to be a spontaneous case (3-6 months) or secondary to a non-solvable condition (may even be indefinite). When using VKA, the optimal international normalized ratio (INR) range is 2 to 3.
- Atrial arrhythmias with high risk of systemic embolism (AF, Atrial Flutter): the indication for chronic anticoagulation in the setting of AF/AFlutter is guided by the risk of embolism. Although the method for the risk estimation varies worldwide, European Society of Cardiology (ESC) guidelines recommend the use of CHADSVASc scale. Generally, it is accepted that the following patients with a score equal to 2 or higher must use chronic oral anticoagulant therapy. For this indication, again, both VKA and DOAC can be used, being the latter the first line therapy. When using VKA, the optimal INR range is 2 to 3.
- Valve heart diseases with high risk of systemic embolism: this situation includes significant mitral stenosis and mechanical valve prosthesis. Tissue valves are not at a high risk of systemic embolism. These situations are the ones with the highest thrombotic risk in the whole area of cardiovascular medicine. As the “power” of the anticoagulation with DOACs was proven to be insufficient in

these patients and entailed an increased thrombotic risk, VKA are the only option. In the case of mitral mechanical prosthesis, the optimal INR range is 2.5-3.5, while aortic valve prosthesis can be handled between 2-3. However, some older prosthetic models are known to be more thrombogenic than newer ones and could require INR of up to 4.

Combined Therapies: SAPT/DAPT with COA

Usually, patients with indication for chronic SAPT with either ASA or clopidogrel and indication for COA are encouraged to take COA alone. This strategy has been assessed is several studies and proved to be safe from a secondary prevention point of view, with a reduced risk of bleeding when compared with concomitant SAPT and COA.

On the other hand, the management of patients with indication for DAPT and COA is normally seen in patients that are undergoing coronary stent implantation and have either atrial arrhythmias (more frequent) or mitral stenosis/mechanical valves (less frequent). Depending on the bleeding risk of the patients, two different approaches can be chosen:

- Normal bleeding risk: short period of triple therapy (1-3 months of DAPT+COA) and a transition period with SAPT and COA (3-12 months). After that, it is recommended to downgrade to simple COA therapy.
- High bleeding risk: the short period is either reduced to 1 month or omitted, leaving the patient on SAPT+COA for a minimum of 12 months.

4. Treatment withdrawal: safety and rationale

It is not the purpose of this paper to give recommendations on timing and indications of withdrawal, but rather explain the changes/effects that the antithrombotic withdrawal entails.

Risks of antiplatelet withdrawal

The main risk of SAPT withdrawal is, of course, the increase in thrombotic risk as explained by a higher chance of MI, stroke or peripheral artery thrombosis. It should be noticed that patients with implanted stents are at a much greater risk of acute events after SAPT withdrawal.

Regarding DAPT, the crucial thing to be understood is that whenever DAPT is used, an abnormally high thrombotic risk underlies. The duration of DAPT regimes depends on several factors, but there should be always a preestablished goal (e.g. 6 months, 12 months, etc.). Transitioning from DAPT to SAPT before the preestablished goal should always be performed under cardiological supervision. Specially, the first month after a coronary stent implantation is of vital importance, as discontinuation of antiplatelet therapy can easily lead to stent thrombosis, a complication that could be fatal.

After discontinuing an antiplatelet agent, the effect does not wear off immediately:

- ASA: 10 days until absence of effect.
- Clopidogrel: 3 days for significant decrease in effect. 5 days for absence of effect.
- Ticagrelor: 3 days for significant decrease in effect. 5 days for absence of effect.
- Prasugrel: 5 days for significant decrease in effect. 7 days for absence of effect.

Risks of anticoagulation withdrawal

Patients with indication for COA that stop taking these medications are at an increased risk for thromboembolic complications. The overall risk of the thrombotic complications highly depends on the indication for COA.

In patients with mitral stenosis or mechanical heart valves, the discontinuation of VKA leads to an extremely thrombotic risk, specially for patients with mechanical valves in mitral position. Thus, discontinuation of this therapy in such patients such always be supervised by cardiovascular professionals and usually requires bridging treatment with heparin (Baumgartner et al., 2017).

On the counterpart, patients without those conditions, can normally be off DOAC/VKA for brief periods of time with an assumable thromboembolic risk, specially in patients with CHADSVASc scores between 2-6. However, discontinuation of VKA is not recommended anymore for procedures other that those with high bleeding risk. After an oral cavity intervention with significant bleeding, DOACs can be safely restarted 24 hours later.

References

Baumgartner, H., Falk, V., Bax, J. J., De Bonis, M., Hamm, C., Holm, P. J., Iung, B., Lancellotti, P., Lansac, E., Rodriguez Munoz, D., Rosenhek, R., Sjogren, J., Tornos

Mas, P., Vahanian, A., Walther, T., Wendler, O., Windecker, S., Zamorano, J. L. & Group, E. S. C. S. D. (2017) 2017 ESC/EACTS Guidelines for the management of valvular heart disease. *European Heart Journal* **38,** 2739-2791. doi:10.1093/eurheartj/ehx391.

European Stroke, O., Tendera, M., Aboyans, V., Bartelink, M. L., Baumgartner, I., Clement, D., Collet, J. P., Cremonesi, A., De Carlo, M., Erbel, R., Fowkes, F. G., Heras, M., Kownator, S., Minar, E., Ostergren, J., Poldermans, D., Riambau, V., Roffi, M., Rother, J., Sievert, H., van Sambeek, M., Zeller, T. & Guidelines, E. S. C. C. f. P. (2011) ESC Guidelines on the diagnosis and treatment of peripheral artery diseases: Document covering atherosclerotic disease of extracranial carotid and vertebral, mesenteric, renal, upper and lower extremity arteries: the Task Force on the Diagnosis and Treatment of Peripheral Artery Diseases of the European Society of Cardiology (ESC). *European Heart Journal* **32,** 2851-2906. doi:10.1093/eurheartj/ehr211.

Ibanez, B., James, S., Agewall, S., Antunes, M. J., Bucciarelli-Ducci, C., Bueno, H., Caforio, A. L. P., Crea, F., Goudevenos, J. A., Halvorsen, S., Hindricks, G., Kastrati, A., Lenzen, M. J., Prescott, E., Roffi, M., Valgimigli, M., Varenhorst, C., Vranckx, P., Widimsky, P. & Group, E. S. C. S. D. (2018) 2017 ESC Guidelines for the management of acute myocardial infarction in patients presenting with ST-segment elevation: The Task Force for the management of acute myocardial infarction in patients presenting with ST-segment elevation of the European Society of Cardiology (ESC). *European Heart Journal* **39,** 119-177. doi:10.1093/eurheartj/ehx393.

Konstantinides, S. V., Torbicki, A., Agnelli, G., Danchin, N., Fitzmaurice, D., Galie, N., Gibbs, J. S., Huisman, M. V., Humbert, M., Kucher, N., Lang, I., Lankeit, M., Lekakis, J., Maack, C., Mayer, E., Meneveau, N., Perrier, A., Pruszczyk, P., Rasmussen, L. H., Schindler, T. H., Svitil, P., Vonk Noordegraaf, A., Zamorano, J. L., Zompatori, M., Task Force for the, D. & Management of Acute Pulmonary Embolism of the European Society of, C. (2014) 2014 ESC guidelines on the diagnosis and management of acute pulmonary embolism. *European Heart Journal* **35,** 3033-3069, 3069a-3069k. doi:10.1093/eurheartj/ehu283.

Neumann, F. J., Sousa-Uva, M., Ahlsson, A., Alfonso, F., Banning, A. P., Benedetto, U., Byrne, R. A., Collet, J. P., Falk, V., Head, S. J., Juni, P., Kastrati, A., Koller, A., Kristensen, S. D., Niebauer, J., Richter, D. J., Seferovic, P. M., Sibbing, D., Stefanini, G. G., Windecker, S., Yadav, R., Zembala, M. O. & Group, E. S. C. S. D. (2019) 2018 ESC/EACTS Guidelines on myocardial revascularization. *European Heart Journal* **40,** 87-165. doi:10.1093/eurheartj/ehy394.

Steffel, J., Verhamme, P., Potpara, T. S., Albaladejo, P., Antz, M., Desteghe, L., Haeusler, K. G., Oldgren, J., Reinecke, H., Roldan-Schilling, V., Rowell, N., Sinnaeve, P., Collins, R., Camm, A. J., Heidbuchel, H. & Group, E. S. C. S. D. (2018) The 2018 European Heart Rhythm Association Practical Guide on the use of non-vitamin K antagonist oral anticoagulants in patients with atrial fibrillation. *European Heart Journal* **39,** 1330-1393. doi:10.1093/eurheartj/ehy136.

Task Force, M., Montalescot, G., Sechtem, U., Achenbach, S., Andreotti, F., Arden, C., Budaj, A., Bugiardini, R., Crea, F., Cuisset, T., Di Mario, C., Ferreira, J. R., Gersh, B. J., Gitt, A. K., Hulot, J. S., Marx, N., Opie, L. H., Pfisterer, M., Prescott, E., Ruschitzka, F., Sabate, M., Senior, R., Taggart, D. P., van der Wall, E. E., Vrints, C. J., Guidelines, E. S. C. C. f. P., Zamorano, J. L., Achenbach, S., Baumgartner, H., Bax, J. J., Bueno, H., Dean, V., Deaton, C., Erol, C., Fagard, R., Ferrari, R., Hasdai, D., Hoes, A. W., Kirchhof, P., Knuuti, J., Kolh, P., Lancellotti, P., Linhart, A., Nihoyannopoulos, P., Piepoli, M. F., Ponikowski, P., Sirnes, P. A., Tamargo, J. L., Tendera, M., Torbicki, A., Wijns, W., Windecker, S., Document, R., Knuuti, J., Valgimigli, M., Bueno, H., Claeys, M. J., Donner-Banzhoff, N., Erol, C., Frank, H., Funck-Brentano, C., Gaemperli, O., Gonzalez-Juanatey, J. R., Hamilos, M., Hasdai, D., Husted, S., James, S. K., Kervinen, K., Kolh, P., Kristensen, S. D., Lancellotti, P., Maggioni, A. P., Piepoli, M. F., Pries, A. R., Romeo, F., Ryden, L., Simoons, M. L., Sirnes, P. A., Steg, P. G., Timmis, A., Wijns, W., Windecker, S., Yildirir, A. & Zamorano, J. L. (2013) 2013 ESC guidelines on the management of stable coronary artery disease: the Task Force on the management of stable coronary artery disease of the European Society of Cardiology. *European Heart Journal* **34,** 2949-3003. doi:10.1093/eurheartj/eht296.
